# Effect of Mechanical Interlocking Damage on Bond Durability of Ribbed and Sand-Coated GFRP Bars Embedded in Concrete Under Chloride Dry–Wet Exposure

**DOI:** 10.3390/polym17060733

**Published:** 2025-03-11

**Authors:** Zhennan Yang, Chunhua Lu, Siqi Yuan, Hao Ge

**Affiliations:** Faculty of Civil Engineering and Mechanics, Jiangsu University, Zhenjiang 212013, China; yangzhennan2022@163.com (Z.Y.); 1000006440@ujs.edu.cn (S.Y.); 2212023025@stmail.ujs.edu.cn (H.G.)

**Keywords:** GFRP bars, bond-slip behavior, chlorine dry–wet exposure, pull-out tests, analytical model, long-term prediction

## Abstract

The substitution conventional steel reinforcement with glass fiber-reinforced polymer (GFRP) bars is a widely adopted strategy used to improve the durability of concrete structures in chloride environments, offering benefits such as enhanced corrosion resistance, reduced maintenance needs, and increased service life. This study investigates the bond behavior between glass fiber-reinforced polymer (GFRP) bars and concrete under long-term chloride dry–wet cycling exposure. Pull-out tests were conducted on various specimens subjected to exposure durations of 0, 3, 6, 9, and 12 months. The experimental results indicate that, after 12 months of chloride dry–wet cycling, the bond strength retention rates of threaded ribbed GFRP with a bond length of 5d, sand-coated GFRP with a bond length of 5d, and threaded ribbed GFRP with a bond length of 7d were 57.9%, 62.2%, and 63.8%, respectively. To predict the GFRP–concrete bond performance after chloride exposure, a novel bond strength model for GFRP bars embedded in concrete, considering the mechanical interlocking effect of ribs, was proposed and validated by the test results. The overall prediction errors for RG-5d, SG-5d, and RG-7d specimens were 0.98, 0.81, and 0.93, respectively. Additionally, a sensitivity analysis was conducted on the main parameters in the model. Finally, the long-term GFRP–concrete bond performance deterioration was estimated using the proposed model. These findings are expected to provide valuable insights into the long-term bond performance and service life prediction of GFRP–concrete members in chloride environments.

## 1. Introduction

Various fiber-reinforced composite materials have been widely used to enhance structural strength, including glass fiber, basalt fiber, carbon fiber, and certain bio-based fibers, all of which exhibit significant potential for further development [1,2,3,4]. Among these, glass fiber-reinforced polymer (GFRP) bars have emerged as a commonly used alternative to traditional steel rebars, particularly in concrete structures exposed to chloride-induced corrosion, due to their excellent corrosion resistance and mechanical properties [5,6].

In reinforced concrete structures, the bond strength between reinforcement and concrete is a critical factor that influences overall structural performance [7]. Unlike steel rebars, FRP bars exhibit anisotropic behavior, and their bond performance is affected by factors such as surface texture and chemical composition [8]. These differences highlight the need for targeted research on the bond behavior of FRP bars, as existing findings on steel–concrete bond performance cannot be directly extrapolated. A comprehensive understanding of FRP–concrete bond properties is essential for the implementation of FRP bars in structural applications [9].

Previous research on the bond performance of FRP bars in concrete has identified several influential factors, including bar diameter, surface texture, and bond length [10,11]. However, as summarized in Table 1, most studies have focused on the bond performance of FRP bars under uncorroded conditions or short-term exposure scenarios, with insufficient attention given to the effects of prolonged chloride exposure [12]. This gap in the literature emphasizes the need for further research on the impact of long-term exposure to aggressive conditions, such as chloride-induced wet–dry cycles, on the bond performance of FRP bars. To evaluate the bond performance of FRP–concrete systems, researchers frequently utilize bond-slip curves derived from pull-out tests. As indicated in Table 1, most existing studies continue to employ traditional mathematical models, which are primarily derived from the development of steel–concrete bonding behavior. While these models provide valuable theoretical insights, they predominantly focus on fitting bond-slip curves rather than analytically describing slip behavior. As a result, the accuracy of these models heavily depends on the quantity and quality of the sample data used for calibration [13]. This reliance on empirical fitting means that deviations in the dataset, variations in material properties, or changes in environmental conditions can significantly impact prediction accuracy. Additionally, these models do not incorporate the long-term degradation effects caused by chloride exposure, which further limits their ability to provide reliable bond strength predictions over time. Therefore, the development of a more precise and physically relevant predictive model is necessary.

The primary objective of this study is to evaluate the bond performance between GFRP bars and concrete under chloride dry–wet cycling exposure and to develop an analytical model that could accurately predict this behavior. The research involved a series of pull-out tests conducted over one year using various GFRP–concrete specimens, which were evaluated at five different exposure intervals. The specimens were categorized based on bond length and surface texture to assess their impact on bond performance as chloride exposure increased. An analytical bond-slip prediction model was also developed, incorporating the damage characteristics of both GFRP bars and concrete. This study provides valuable insights into critical factors affecting bond strength, such as surface texture, bond length, and exposure duration, while proposing a new approach to predict bond-slip behavior under long-term exposure conditions.

## 2. Experimental Setup

**Figure 1:**
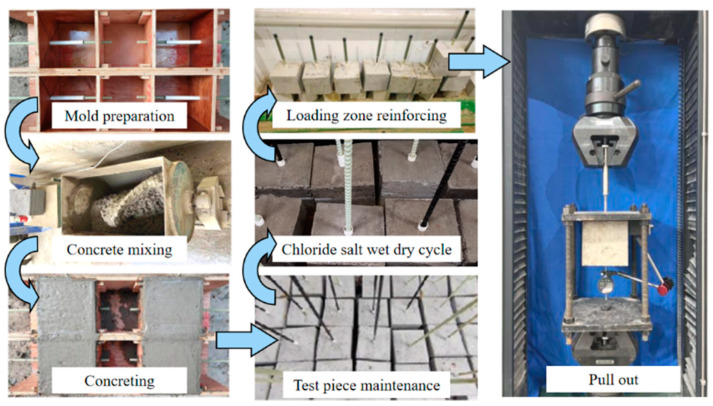
A flow diagram to illustrate the testing processes.

Figure 1 shows the key testing steps in the experimental procedure. The testing process begins with mold preparation, followed by concrete mixing, and then casting to produce the test specimens. After casting, the specimens undergo 28-day curing to achieve the desired material properties. Once the curing phase is completed, the specimens are subjected to chloride salt wet–dry cycles, simulating aggressive environmental conditions to assess the long-term effects of chloride exposure. Following the exposure phase, prior to pull-out tests, the loading zone is reinforced using steel tubes to prevent damage to the GFRP bars by the loading grip. Finally, the specimens undergo the pull-out test, where the bond performance between the GFRP bar and concrete is evaluated under controlled loading conditions.

### 2.1. Design of Pull-Out Specimen

**Figure 2:**
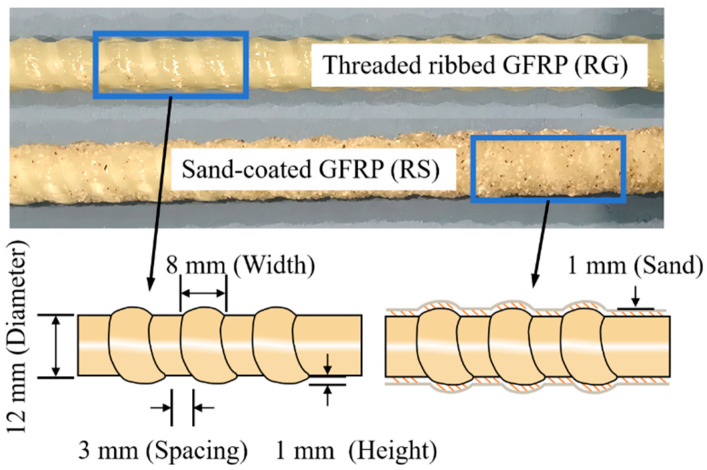
Geometric details of the GFRP bars.

The experimental investigation utilized two types of 12 mm diameter GFRP bars: SG bars with a sand-coated surface and RG bars with a threaded ribbed surface. The key geometric parameters of the ribbed bars, including rib spacing, width, and height, are illustrated in Figure 2. Both GFRP bar types were manufactured by Jiangsu Feibo New Material Technology Co. (Yancheng, China, state for China), with a tensile strength of 874.1 MPa and an elastic modulus ($E_{f}$) of 36 GPa. The ribs were produced by winding a strand tightly around the smooth GFRP bundles during the manufacturing process, resulting in a helical groove pattern on the bar surface. As listed in Table 2, the conventional C40 concrete used for casting the specimens achieved a 28-day compressive strength of 47.1 MPa.

**Figure 3:**
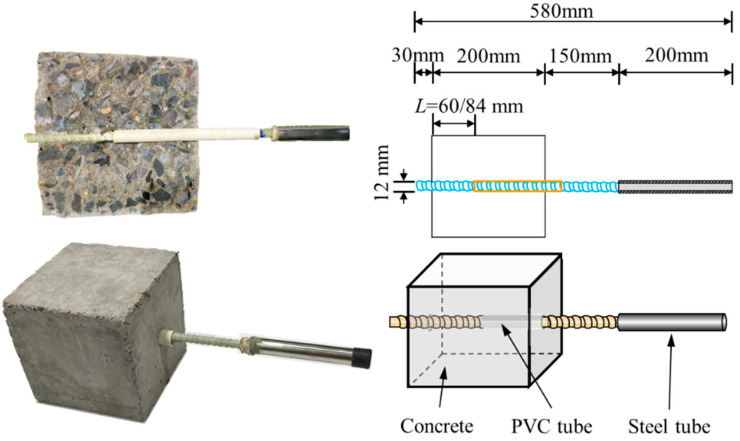
Pull-out test specimen diagram.

A series of pull-out test specimens were fabricated to evaluate the bond behavior between GFRP bars and conventional concrete. As shown in Figure 3, GFRP bars were cut to a length of 580 mm and concentrically embedded in concrete cubes measuring 200 mm × 200 mm × 200 mm, following the guidelines of ACI 440.3R-12. A 16 mm diameter PVC pipe served as a bond breaker to control the bond length. To prevent GFRP bar rupture during testing, a steel tube, 200 mm long, was bonded to the loaded end of the bar using epoxy adhesive. During casting, the GFRP bars were oriented perpendicular to the specimen surface to minimize micro-void formation at the bar-concrete interface. The molds were removed 24 h after casting, and the specimens were cured under ambient conditions for 28 days. The embedded length of the bars in the pull-out specimens was set to five times the bar diameter (5*d*). To further investigate the effect of embedment length, an additional set of specimens was cast with an embedded length equivalent to seven times the bar diameter (7*d*). In addition, a certain number of 150 mm cubic blocks were also prepared to assess the compressive strength variation during the dry–wet cycling test.

### 2.2. Chloride Dry–Wet Exposure Program

**Figure 4:**
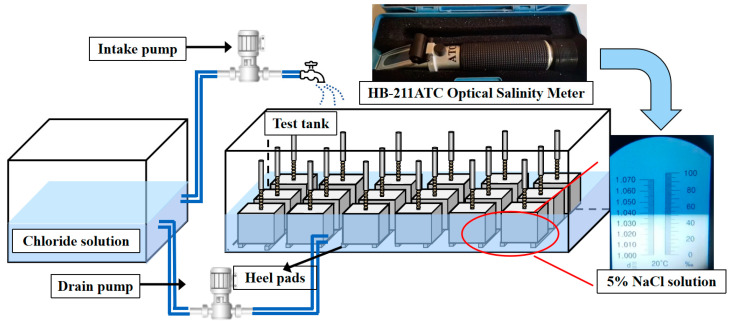
Automated chloride dry–wet cycle exposure device and chlorine concentration monitoring.

Following 28 days of natural curing, the pull-out specialties were subjected to wet–dry cycling in solution, and a 5% NaCl solution was chosen to approach the salinity of seawater more closely [14]. Each cycle comprised a 12 h wet phase followed by a 12 h dry phase, with an average temperature of 20 ± 2 °C and a relative humidity of 80% to simulate marine conditions [15]. During exposure, the specimens were positioned vertically on heel blocks, allowing for chloride diffusion in the concrete from the free end. As shown in Figure 4, the TR-ZXC artificial marine tidal salt spray environmental control system, manufactured by Shanghai Tongrui Instrument Equipment Co. (Shanghai, China, state for China), was used to regulate the cyclic exposure time and temperature of artificial seawater during chlorine exposure. Additionally, the HB-211ATC Optical Salinity Meter (Bestone Industrial Ltd., Shenzhen, China) was employed to monitor chloride ion concentration, ensuring the consistency and accuracy of the exposure conditions.

To assess the degradation of GFRP–concrete bond performance, due to chloride exposure, the pull-out tests were conducted at five intervals: immediately after curing (0 months), and at 3, 6, 9, and 12 months after chloride exposure. The three groups of pull-out specimens were fabricated and tested, as summarized in Table 3. As given in Table 3, the specimen identifier comprises three components: the initial letters denote the GFRP bar surface texture, the subsequent numeral indicates the effective bond length as a multiple of the bar diameter, and the final numeral specifies the duration of chloride exposure in months. For example, SG-5d-6 refers to a specimen with a sand-coated GFRP bar, an effective bond length of five times the bar diameter, and six months of chloride exposure.

### 2.3. Pull-Out Loading Scheme

**Figure 5:**
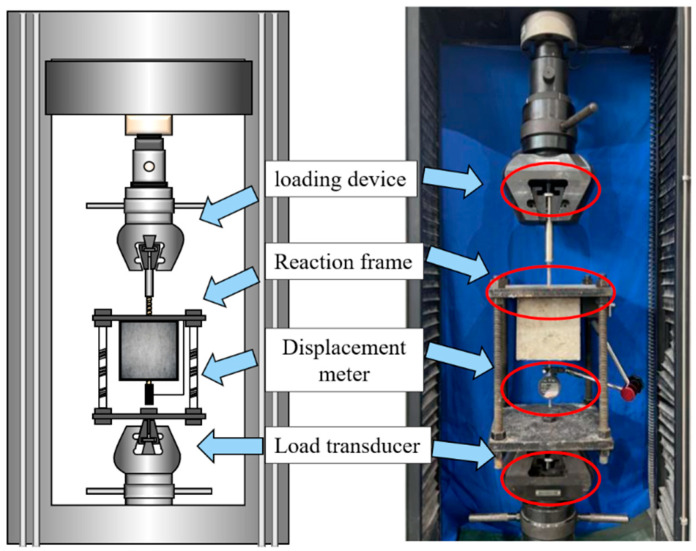
Loading device for the uniaxial pull-out test.

This study followed the guidelines established by ACI 440.3R-12 and CSA S806-12 for the design of test specimens and the experimental setup. As illustrated in Figure 5, pull-out tests were performed using a 600 kN capacity servo-hydraulic testing machine manufactured by Shenzhen SUNS Technology Stock Co. (Shenzhen, China, state for China). The force measurement accuracy of the testing machine is ±1%, ensuring reliable data acquisition. Additionally, a hydraulic pressure sensor was utilized for force measurement and control during the tests. The specimens were securely positioned on a steel frame connected to the top grip system. To minimize misalignment and reduce friction between the concrete cube and the steel plate, a soft rubber pad and a low-friction film were employed. A displacement-controlled loading scheme was utilized to capture the complete post-peak behavior of the specimens. The load was applied to the GFRP bar at a rate of 1 mm/min until failure occurred (ACI 440.3R-12). The machine system could precisely record the applied load and displacement during the test, while an extra LVDT was utilized to measure the displacement at the free end of the tested GFRP bar. The LVDT has a measurement accuracy of 0.01 mm and an error margin of 1%, providing precise and consistent displacement data throughout the experiment.

The bond behavior discussed in this paper is characterized by nominal bond stress (τ) calculated using the following equation:

(1) $$\tau =\frac{F_{test}}{\pi dL}$$

where $F_{test}$ is the applied load during the test; d is the nominal bar diameter; L is the bond length, which is 5*d* or 7*d* in this test.

## 3. Experimental Results and Discussions

### 3.1. Failure Patterns and Test Results

**Figure 6:**
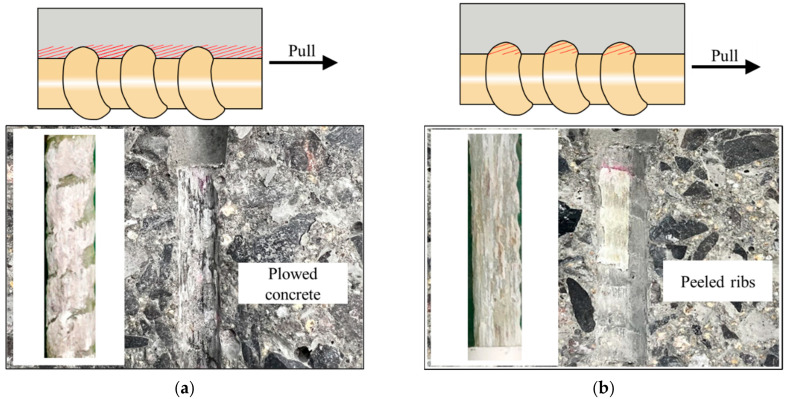
Typical failure patterns of tested pull-out specimens: (**a**) Failure A pattern; (**b**) Failure B pattern.

Two distinct failure patterns were observed in the pull-out tests: Type A (pull-out failure) and Type B (peeling-off failure). In Type A failure (Figure 6a), residual concrete remained between the ribs of the extracted GFRP bar, indicating that the bond strength was primarily maintained by mechanical interlock. As the applied load increased, stress transferred through the ribs, and when the bond strength was exceeded, the GFRP bar was extracted along with residual concrete. In contrast, Type B failure (Figure 6b) exhibited extensive rib wear, with no concrete residue left on the bar surface, suggesting that damage was concentrated on the ribs, leading to progressive material loss and eventual peeling-off failure.

The red area in Figure 6 marks the weak zone that experienced the most damage, where bond failure initiated and propagated. The occurrence of these failure modes was influenced by the relative strengths of the GFRP bar and concrete. Type A failure was more likely when the concrete strength was higher, ensuring strong mechanical interlock, while Type B failure tended to occur when the GFRP rib strength was lower, leading to excessive wear and peeling. Additionally, environmental factors such as chloride ingress can alter the failure mechanism over time, potentially causing a transition from Type A to Type B.

The bond-slip performance and corresponding failure patterns are summarized in Table 4. With the exception of the RG-5d-0 specimen (which underwent no chloride exposure) and some RG-7d specimens (which were subjected to shorter exposure durations), all other specimens exhibited a Type B failure. This finding suggests that the ribs of the GFRP bars deteriorated significantly after chloride dry–wet exposure. Furthermore, the tested peak bond strength ($\tau_{t}$) displayed a general decreasing trend with the increasing duration of chloride exposure. However, variations in slip corresponding to the peak point ($s_{t}$) differed significantly among test specimens.

Significant differences in bond strength were observed between the two rebar types under identical bond lengths and chloride exposure durations. Initially, the RG-5d-0 specimen exhibited a peak bond strength of 36.8 MPa, while the SG-5d-0 specimen recorded a lower value of 21.7 MPa. After 12 months of chloride exposure, the bond strength decreased to 21.3 MPa for the RG-5d-12 specimen and to 13.5 MPa for the SG-5d-12 specimen. Conversely, the corresponding slip displacement was slightly higher in the RG-type GFRP specimens. For instance, RG-5d-0 exhibited a slip of 5.8 mm compared to 4.7 mm for SG-5d-0. The SG-5d specimens displayed minimal changes in slip with chloride exposure, with slip values of 4.7 mm for SG-5d-0 and 4.9 mm for SG-5d-12. Additionally, an increase in bond length generally resulted in a decrease in peak bond strength. For example, the RG-5d-0 specimen had a peak bond strength of 36.8 MPa, whereas the RG-7d-0 specimen exhibited a lower value of 29.0 MPa. Longer bond lengths were also associated with greater slip displacements; RG-7d-0 exhibited a slip of 10.7 mm compared to 5.8 mm for RG-5d-0.

In summary, peak bond strength was affected by chloride exposure duration, surface texture, and effective bond length. While the corresponding slip displacement did not follow a consistent pattern across all specimens, it was generally higher in those with increased bond lengths and RG-type GFRP bars. These results highlight the complex interactions governing bond–slip behavior under different exposure conditions, which is further examined in subsequent sections.

### 3.2. Effects of Surface Textures

**Figure 7:**
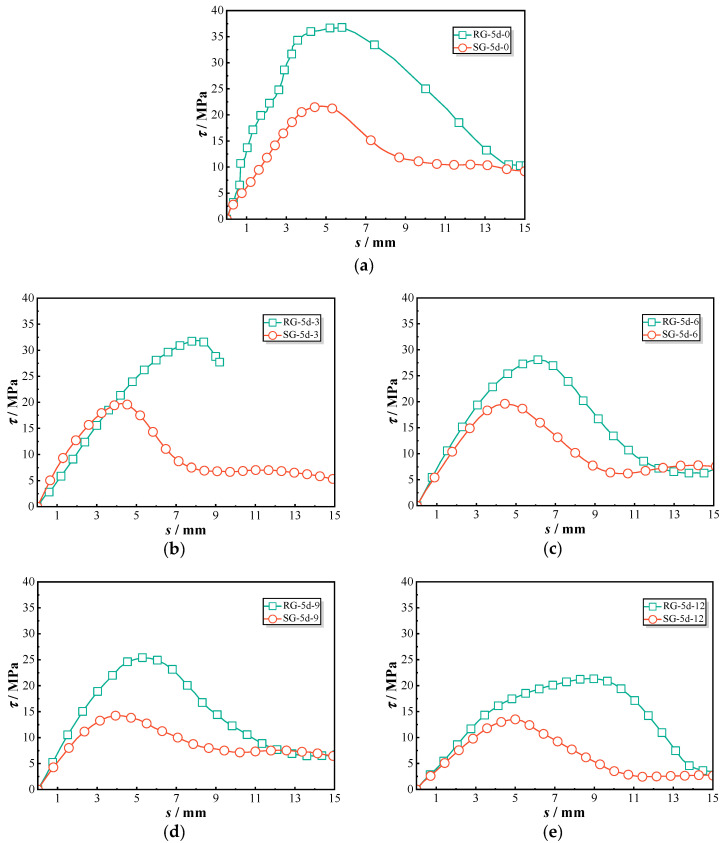
Bond–slip curves of tested RG-5d and SG-5d specimens: (**a**) RG-5d-0 and SG-5d-0; (**b**) RG-5d-3 and SG-5d-3; (**c**) RG-5d-6 and SG-5d-6; (**d**) RG-5d-9 and SG-5d-9; (**e**) RG-5d-12 and SG-5d-12.

Figure 7 provides a detailed comparison of the bond–slip behavior of threaded ribbed GFRP (RG) bars and sandblasted GFRP (SG) bars. The bonding behavior of each group was similar, and one representative curve was selected for discussion. Under initial conditions without chloride exposure, specimen RG-5d-0 exhibited a significantly steeper ascending slope, reaching a peak bond strength of 36.8 MPa. In contrast, the SG-5d-0 bars displayed lower bond strength during this phase, with a more gradual increase in bond stress. As the duration of chloride exposure increased from 0 to 12 months, both RG-5d and SG-5d specimens showed a decline in bond strength. However, RG-5d specimens consistently exhibited a steeper ascending slope and higher peak values compared to SG-5d specimens throughout the chloride exposure period.

**Figure 8:**
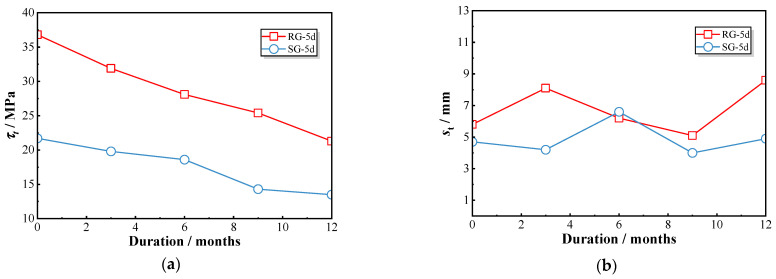
Variations in peak bond strength and the corresponding slip of RG-5d and SG-5d specimens: (**a**) peak bond strength; (**b**) corresponding slip.

Figure 8 illustrates the peak bond strength and corresponding slip values. For RG-5d series specimens, the bond stress decreased from 36.8 MPa at 0 month to 21.3 MPa at 12 months, resulting in a retention ratio of 0.58. In comparison, the sand-coated GFRP bars (SG-5d) showed a reduction from 21.7 MPa to 13.5 MPa over the same period, corresponding to a retention ratio of 0.62. The peak strength of the RG-5d specimens was approximately 1.4 times higher than that of the SG-5d specimens during all exposures, indicating that both types experienced a comparable rate of deterioration. However, no consistent pattern of change was observed in the slip values at peak points. In general, the RG-5d specimens exhibited greater slip compared to the SG-5d specimens, suggesting that sand-coated GFRP bars demonstrate lower stiffness.

The bond performance differences between threaded ribbed GFRP (RG) bars and sand-coated GFRP (SG) bars stem from their distinct bonding mechanisms with the surrounding concrete matrix. RG bars, with their continuous helical ribs, create a strong mechanical interlock, allowing for more efficient stress transfer and improved shear resistance along the bond interface [16]. The ribbed surface also enhances confinement in the surrounding concrete, reducing slip and improving pull-out resistance. Additionally, as chloride exposure progresses, the rougher profile of RG bars helps maintain bond integrity, providing better bond durability compared to SG bars [17].

In contrast, SG bars rely primarily on friction and adhesion, with the sand-coated layer reducing mechanical interlocking efficiency. While the roughened sand surface initially provides high frictional resistance during the early slip stage, the peak bond strength of SG bars is lower than that of RG bars. Studies have shown that the sand layer can detach under cyclic loading or sustained exposure to aggressive environments, decreasing the effective contact area at the bar–concrete interface [18]. This leads to a more rapid decline in bond strength, particularly in chloride-rich conditions where aggressive ion ingress weakens interfacial adhesion. Based on the results of this study, SG specimens consistently exhibited lower bond performance than RG specimens, regardless of chloride exposure duration. This finding indicates that, under the specific conditions of this experiment, the ribbed structure significantly enhances the bond behavior of GFRP bars in chloride-exposed environments.

### 3.3. Effects of Bond Length

**Figure 9:**
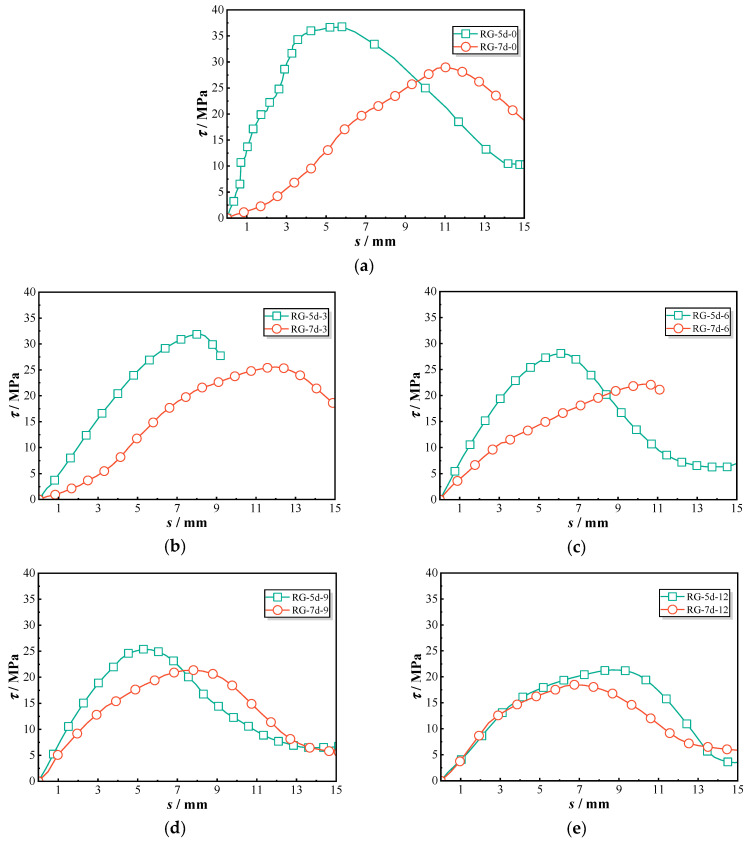
Bond–slip curves of tested RG-5d and RG-7d specimens: (**a**) RG-5d-0 and RG-7d-0; (**b**) RG-5d-3 and RG-7d-3; (**c**) RG-5d-6 and RG-7d-6; (**d**) RG-5d-9 and RG-7d-9; (**e**) RG-5d-12 and RG-7d-12.

Figure 9 presents a detailed comparison of the bond–slip behavior for GFRP bars with different bond lengths (5*d* and 7*d*). For specimens without chloride exposure, RG-5d-0 exhibited a steeper increase in average bond stress, resulting in higher average bond strength, while RG-7d-0 demonstrated greater slip displacement. These results suggest that shorter bond lengths enhance average bond strength.

With increased chloride exposure duration, both RG-5d and RG-7d specimens showed significant reductions in bond strength. Over time, the bond–slip curves for the two bond lengths converged, indicating that the influence of bond length diminished under prolonged chloride exposure.

**Figure 10:**
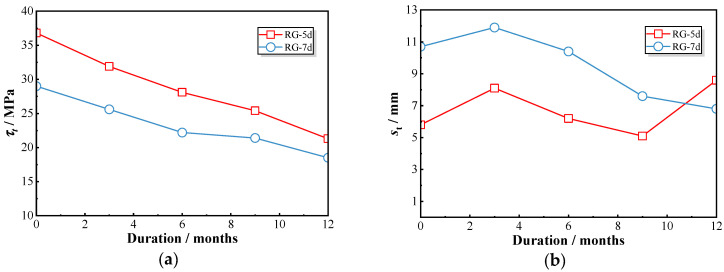
Variations in peak bond strength and the corresponding slip of RG-5d and RG-7d specimens: (**a**) peak bond strength; (**b**) corresponding slip.

The analysis of peak bond strength and the corresponding slip is presented in Figure 10. A distinct difference in peak bond stress was observed between the RG-5d-0 and RG-7d-0 specimens, with RG-5d-0 exhibiting a higher peak bond stress of 36.8 MPa, compared to 29.0 MPa for RG-7d-0—a 21% reduction. Additionally, the slip displacement for RG-7d-0 was approximately 80% greater than that of RG-5d-0. After 12 months of chloride exposure, RG-5d-12 showed only a 15% higher peak bond stress than RG-7d-12; however, their slip displacements were more comparable, with RG-5d-12 being 26% greater than RG-7d-12.

This difference can be primarily attributed to the uneven stress distribution along the GFRP bar in the pull-out specimens. Under direct tensile loading, asynchronous deformation may occur between the core and outer layers of the ribbed GFRP bar, leading to uneven stress distribution across the cross-section and shear lag [19]. During the pullout test, the bond stress along the rebar was not uniformly distributed, and the ultimate bond stress gradually migrated from the loaded end to the free end. When the bond length is short, the high-stress zone expands proportionally, causing the stress curve to bulge more prominently along the anchorage length. This results in a higher average bond stress. Conversely, when the bond length increases, the bond stress distribution becomes more uniform, leading to a lower average bond stress [20].

### 3.4. Effects of Chlorine Salt Erosion

**Figure 11:**
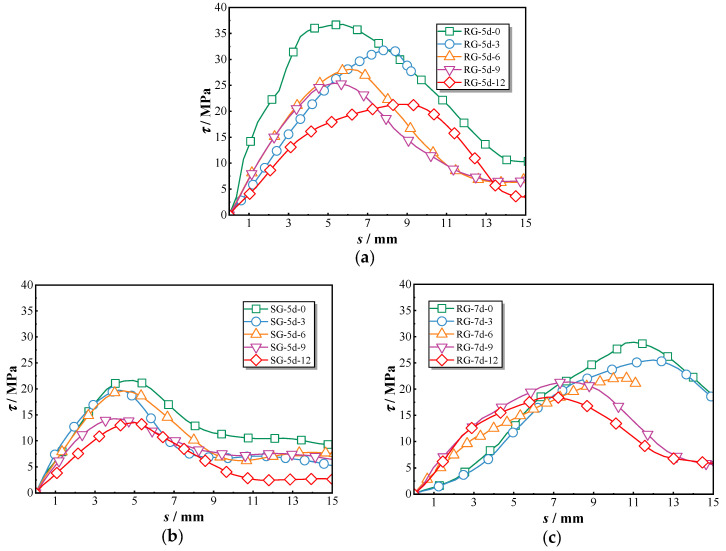
Bond–slip curves of all tested specimens: (**a**) RG-5d group; (**b**) SG-5d group; (**c**) RG-7d group.

Figure 11a illustrates the bond–slip variations of RG-5d specimens under different chloride exposure durations. The RG-5d-0 specimen initially exhibited a steep ascending slope, reflecting strong bond strength. However, as chloride exposure duration increased, the slope became less pronounced. Figure 11b demonstrates the bond–slip behaviors of SG-5d specimens, which experienced little changes in bond performance during the first six months of exposure, followed by a reduction in curve convexity after nine months. In Figure 11c, the bond–slip behaviors of RG-7d specimens reveal two distinct trends. Before nine months of exposure, the bond–slip curve exhibits an S-shaped trajectory characterized by a gradual increase during the initial slip phase, a sharper rise around the 3 mm slip, peaking at approximately 6.5 mm. This pattern results in a higher peak slip displacement. After nine months, the curve adopts a different shape, with a steep initial slope followed by a pronounced reduction at the inflection point, resulting in lower peak slip displacement and bond strength. Overall, chloride exposure significantly impacts the pull-out behaviors of GFRP–concrete bond specimens. As exposure time increases, the bond–slip curves shift from a convex shape to a more flattened profile, reflecting the gradual deterioration in bond strength.

**Figure 12:**
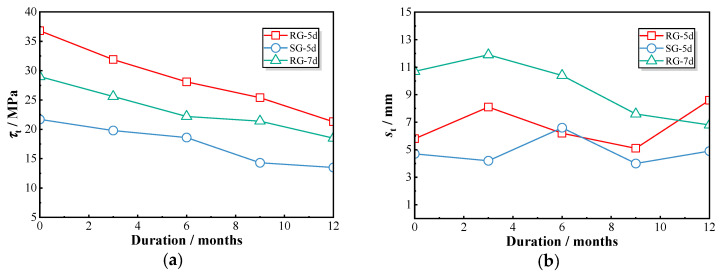
Variations in peak bond strength and the corresponding slip of all tested specimens: (**a**) peak bond strength; (**b**) corresponding slip.

Figure 12a illustrates the progressive decline in peak bond strength for each group of specimens subjected to chloride exposure. Specifically, the peak bond strength of the RG-5d series decreased from 36.8 MPa (RG-5d-0) to 21.3 MPa (RG-5d-12), the SG-5d series decreased from 21.7 MPa (SG-5d-0) to 13.5 MPa (SG-5d-12), and the RG-7d series decreased from 29.0 MPa (RG-7d-0) to 18.5 MPa (RG-7d-12). This consistent decline across all groups indicates that chloride exposure induces a comparable rate of bond strength deterioration in GFRP–concrete pull-out specimens, irrespective of the specimen type. Figure 12b presents the slip values corresponding to the peak point under chloride exposure. While the RG-5d and SG-5d groups display a fluctuating pattern without a clear trend, the RG-7d group shows a significant variation in peak slip around the 9-month exposure period. For example, the peak slip of the RG-7d-6 specimen reached 10.4 mm but then decreased to 7.6 mm for the RG-7d-9 specimen, representing a reduction of 26.9%. Prolonged exposure thus results in a noticeable reduction in peak slip values for the RG-7d specimens.

As previously discussed, the pull-out behavior of GFRP–concrete bond specimens undergoes considerable changes with the increasing duration of chloride dry–wet cycles. Chloride ingress weakens the bond between the GFRP bars and the concrete, resulting in a shift in the bond–slip curves from a convex shape to a flattened trend [21]. All specimen groups exhibited a gradual decline in peak bond strength at a similar rate over the exposure period. However, the slip values of the RG-5d and SG-5d groups showed a wave-like pattern, whereas the RG-7d group displayed significant changes around the 9-month exposure mark, likely due to alterations in the failure mode of the specimens.

**Figure 13:**
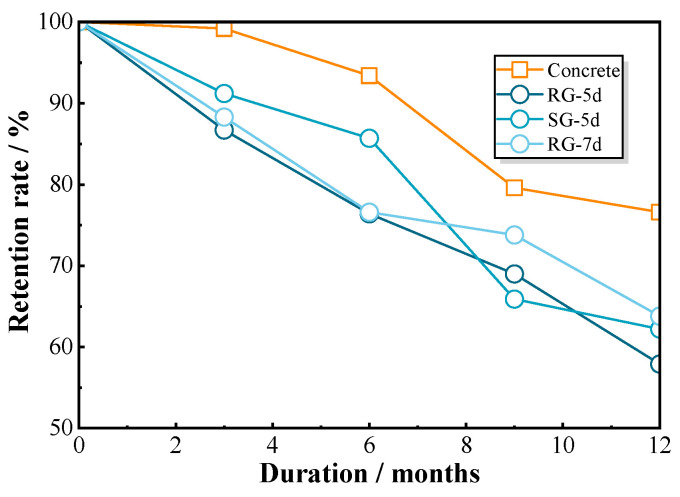
Comparison of bond strength with concrete compressive strength in retention rate.

To assess the impact of concrete strength on bond–slip behavior under chloride exposure, the compressive strength of concrete exposed to the same environment was tested. Figure 13 compares the retention rates of concrete compressive strength and bond strength over time. The data revealed that the reduction in concrete compressive strength does not directly correlate with the degradation of bond strength in GFRP bars. While the concrete retained over 90% of its initial strength during the first four months, it dropped sharply to 76.6% after 12 months of exposure. In contrast, the bond strength of the RG-5d specimens steadily declined by 42.1% over the same period. This suggests that the reduction in concrete strength alone does not account for the observed decrease in bond strength [22,23]. The degradation of ribbed FRP bars in chloride environments appears to play a decisive role in bond strength deterioration [24].

The experimental results of this study were compared with previous research on the bond behavior of GFRP bars under seawater exposure. Mai et al. conducted a 9-month seawater exposure test using artificial seawater with a salinity of 3% and observed a bond strength reduction of 17.5%, which aligns with the findings of this study, as both indicate a decline in bond strength after exposure. Similarly, Pan et al. reported a 39.68% reduction in bond strength after 6 months of seawater exposure, demonstrating a more significant degradation. However, their study also included mechanical loading during exposure, which may have contributed to the greater bond strength reduction compared to this study [25].

In contrast, Nepomuceno et al. reported an increase of 39% in bond strength after one year of seawater exposure, which contradicts the findings of this study. However, separate strength tests on GFRP bars alone in their study indicated that the GFRP material itself experienced degradation, suggesting that the bond strength increase could be attributed to enhanced cement hydration in the presence of seawater, rather than an improvement in the GFRP–concrete interface [26].

The results of studies that reported bond strength reduction are consistent with the findings of this study and can be attributed to seawater-induced damage to both GFRP bars and concrete. On the other hand, studies reporting bond strength improvement suggest that the chemical interaction between seawater and cementitious materials may enhance hydration, leading to a temporary increase in bond strength. These comparisons highlight the complex influence of seawater exposure on GFRP–concrete bonding and the need for further investigation to fully understand the long-term degradation mechanisms [27].

## 4. Theoretical Modeling

### 4.1. Analytical Model for Bond Stress and Slip Behavior

**Figure 14:**
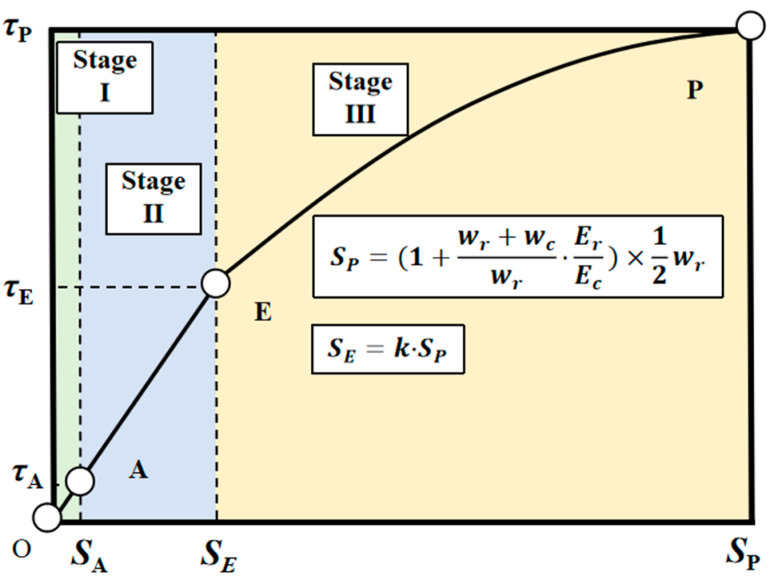
Three stages for the ascending portion in the bond–slip curve.

According to the test descriptions in Section 3, chloride exposure significantly influenced the failure pattern and bond strength of GFRP–concrete pull-out specimens. Therefore, it is essential to develop a mechanical model for the bond–slip behavior of GFRP bars in concrete that accounts for chloride-induced damage. After considering that practical applications often prioritize the peak strength, this paper emphasized the characteristics of the ascending segment of the bond–slip curve. As illustrated in Figure 14, the ascending segment can be typically divided into three distinct stages based on their contributions to bond stress [28]. In Stage I, bond stress is primarily attributed to adhesion ($F_{a}$), friction ($F_{f}$), and mechanical interlock ($F_{m}$). In Stage II, the bond–slip behavior causes a transition from static to dynamic friction, rendering the adhesive effect negligible. In Stage III, the mechanical interlock behavior enters an inelastic phase due to the plastic deformation of the ribs.

**Figure 15:**
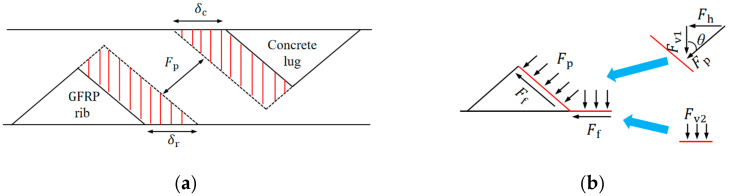
Schematic diagram illustrating slip generation and force generation on a single rib: (**a**) slip generation; (**b**) force generation.

Since Stage I constitutes a relatively small portion of the entire slip process, the bond–slip model can be effectively calculated and analyzed from Stage II onward. In Stages II and III, the slip behavior of the bars is attributed to a combination of deformations in both the concrete and the GFRP ribs. Given that the rib width ($w_{r}$) is typically much larger than the rib height ($h_{r}$), the ribs can be approximated as triangular shapes on the bar [29]. As illustrated in Figure 15a, the overall slip of the GFRP bar (s) can be determined as follows:

(2) $$\delta_{c}=\frac{w_{r}+w_{c}}{w_{r}}\cdot \frac{E_{r}}{E_{c}}\delta_{r}$$

(3) $$s=(1+\frac{w_{r}+w_{c}}{w_{r}}\cdot \frac{E_{r}}{E_{c}})\delta_{r}$$

where $\delta_{c}$ and $\delta_{r}$ are the axial deformation in concrete and GFRP ribs in Figure 15a, respectively; $w_{r}$ is the width of the GFRP rib; and $w_{c}$ is the width between the ribs.

Since GFRP bars are anisotropic materials, their transverse elastic modulus is typically lower than their longitudinal elastic modulus [30]. Therefore, in this study, the transverse modulus of elasticity for GFRP ribs is denoted as $E_{r}$, with γ representing the discount factor applied to the longitudinal modulus of elasticity $E_{f}$.

In Type B failure, the ribs of the GFRP bar are nearly completely worn away, while the concrete remains largely intact. Consequently, the concrete can be considered a rigid body during the pull-out test to analyze the elastic deformation of the rib, but in fact, concrete also undergoes deformation simultaneously. At the peak of Type B failure, it is assumed that the rib width is compressed by the concrete lug to half of its original value, a hypothesis widely applied in bond–slip analysis at this scale [29,31]. Therefore, the slip corresponding to the peak bond strength ($s_{P}$) can be calculated as follows:

(4) $$s_{P}=(1+\frac{w_{r}+w_{c}}{w_{r}}\cdot \frac{E_{r}}{E_{c}})\times \frac{1}{2}w_{r}$$

A parameter k is introduced to determine point *E* for the limit state of Stage II:

(5) $$s_{E}=k\cdot s_{P}=(1+\frac{w_{r}+w_{c}}{w_{r}}\cdot \frac{E_{r}}{E_{c}})\times \frac{k}{2}w_{r}$$

**Figure 16:**
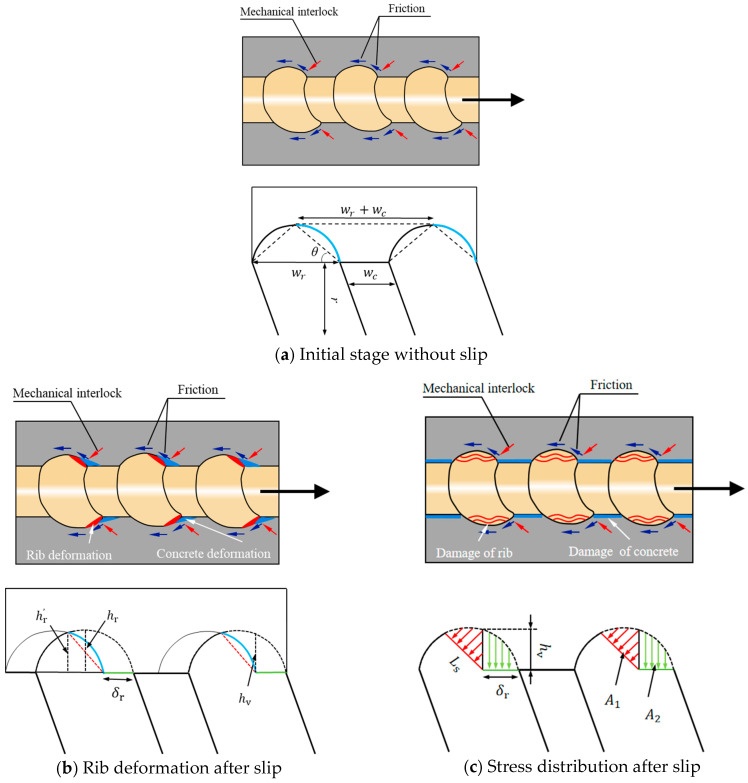
Force diagram of deformed GFRP bars: (**a**): Initial stage without slip; (**b**): Rib deformation after slip; (**c**) Stress distribution after slip.

During these stages, bond stress is primarily influenced by the mechanical interlock between the FRP ribs and the concrete lugs. A simplified model is proposed in Figure 16 to illustrate the compressive deformation behavior of FRP ribs, suggesting that damage to ribbed FRP bars is primarily caused by the compressive crushing of ribs rather than direct slip failure at the bar surface. Figure 16a illustrates the initial state of a GFRP bar under pull-out loading, where the original blue semicircular rib is compressed by the concrete lugs, with specific geometric characteristics marked. Figure 16b shows the state of the FRP bars after experiencing rib deformation by $\delta_{r}$. The deformed contact profile is divided into two components: the deformed rib (blue) and the extruded horizontal Section (green). For simplification, the blue curve is approximated as the red dotted line. Figure 16c depicts the stress distribution following rib deformation, where the red area represents compressive stress on the deformed rib, and the green area indicates radial stress exerted on the GFRP bar. Among these two regions, only the red area generates a longitudinal component force that contributes to pull-out resistance, while the green area induces radial force, enhancing the frictional effect along the GFRP bar surface.

As depicted in Figure 16c, the length of the contact surface of the FRP rod after rib compression deformation is represented by $L_{s}$, while the maximum radial compression deformation is denoted as $h_{v}$. Both parameters can be determined using the following equations:

(6) $$L_{s}=\frac{w_{r}-\delta_{r}}{2cos\theta }$$

(7) $$h_{v}=h_{r}-h_{r}^{\prime }$$

The radial deformation on the contact surface changes linearly along the longitudinal direction of the FRP bar. Given that the vertical radius of the GFRP bar (r) is typically much greater than the rib height ($h_{r}$), the average vertical strain ($\varepsilon_{v}$) on the contact surface can be determined as follows:

(8) $$\varepsilon_{v}=\frac{h_{v}/2}{r}$$

The average radial stress on the contact surface can be derived through Equation (9):

(9) $$\sigma_{v}=E_{r}\varepsilon_{v}$$

Then, the vertical forces in Figure 15b ($F_{v1}$ and $F_{v2}$) generated by a single rib can be calculated as:

(10) $$F_{v1}=2\pi rL_{s}\sigma_{v}$$

(11) $$F_{v2}=2\pi r\delta_{r}\sigma_{v}$$

The horizontal force generated at the contact surface is:

(12) $$F_{h}=F_{v1}tan\theta$$

The friction force $F_{f}$ caused by rib compression deformation can be obtained as:

(13) $$F_{f}=u_{s}F_{v1}tan\theta +u_{s}F_{v2}=u_{s}F_{h}+u_{s}F_{v2}$$

where $u_{s}$ denotes the friction coefficient of the GFRP rib.

Then, the bond stress along the GFRP bars at unit length ($w_{r}+w_{c}$) can be obtained:

(14) $$\tau =\frac{F_{h}+F_{f}}{2\pi r(w_{r}+w_{c})}$$

Hence, the bond stress for stages I and II (from point O to point E) can be collated as:

(15) $$\tau_{OE}=\left(1+u_{s}\right)E_{r}\frac{h_{r}}{rw_{r}(w_{r}+w_{c})}\frac{sin\theta }{2{cos}^{2}\theta }\delta_{r}\left(w_{r}-\delta_{r}\right)+u_{s}E_{r}\frac{h_{r}}{rw_{r}s_{r}}{\delta_{r}}^{2}$$

In stage III, the friction remains constant at the maximum friction force observed in stage II. Another way of saying this is that the friction force of stage E~P is constant as the friction force at point E, denoted as $\tau_{Ef}$:

(16) $$\tau_{Ef}=\tau_{E}-\gamma E_{r}\frac{h_{r}}{rw_{r}(w_{r}+w_{c})}\frac{sin\theta }{2{cos}^{2}\theta }\delta_{A}\left(w_{r}-\delta_{E}\right)$$

Based on this, the bond strength for stage III (from point E to point P) can be collated as:

(17) $$\tau_{EB}=E_{r}\frac{h_{r}}{rw_{r}(w_{r}+w_{c})}\frac{sin\theta }{2{cos}^{2}\theta }[\delta_{r}\left(w_{r}-\delta_{r}\right)-\frac{k}{2}w_{r}(w_{r}-\frac{k}{2}w_{r})]+\tau_{Ef}$$

This section presents an analytical model for predicting bond–slip behavior in FRP-concrete pull-out interactions. In the model, the slip is presented in Equation (3), while the bond stresses for the OE segment and the EB segment are shown in Equations (15) and (17), respectively.

$$\left\{\begin{array}{c} s=(1+\frac{w_{r}+w_{c}}{w_{r}}\cdot \frac{E_{r}}{E_{c}})\delta_{r}\\ \tau =\left\{\begin{array}{cc} \left(1+u_{s}\right)E_{r}\frac{h_{r}}{rw_{r}(w_{r}+w_{c})}\frac{sin\theta }{2{cos}^{2}\theta }\delta_{r}\left(w_{r}-\delta_{r}\right)+u_{s}E_{r}\frac{h_{r}}{rw_{r}s_{r}}{\delta_{r}}^{2} & s<s_{E}\\ E_{r}\frac{h_{r}}{rw_{r}(w_{r}+w_{c})}\frac{sin\theta }{2{cos}^{2}\theta }[\delta_{r}\left(w_{r}-\delta_{r}\right)-\frac{k}{2}w_{r}(w_{r}-\frac{k}{2}w_{r})]+\tau_{Ef} & s_{E}\leq s \end{array}\right. \end{array}\right.$$

The resulting bond stress is uniformly distributed across the effective bond zone, while the calculated slip represents the cumulative displacement of the FRP bars. Furthermore, the model is applicable to specimens with a failure mode of peeling off, and this model does not account for the effects of chloride exposure.

### 4.2. Model Modification Under Chloride Exposure

Previously, Section 4.1 presented an analytical model for bond–slip behavior without considering the effects of chloride exposure. However, as demonstrated in Section 3, the experimental results show that chloride wet–dry cycles significantly impact the bond performance of GFRP–concrete specimens. Both GFRP bars and concrete can deteriorate under these conditions, indicating the need to revise the analytical model for more accurate long-term performance predictions. To address this, two damage indicators denoted as $d_{r}$ and $d_{c}$ are introduced to quantify the deterioration in the elastic modulus of GFRP bars and the compressive strength of concrete, respectively. In this study, $d_{r}$ and $d_{c}$ account solely for the effects of chloride wet–dry cycles. Assuming uniform damage progression, the two indicators can be calculated as follows:

(18) $$d_{r}=\frac{E_{r}-E_{r}^{\prime }}{E_{r}}$$

(19) $$d_{c}=\frac{f_{c}-f_{c}^{\prime }}{f_{c}}$$

**Figure 17:**
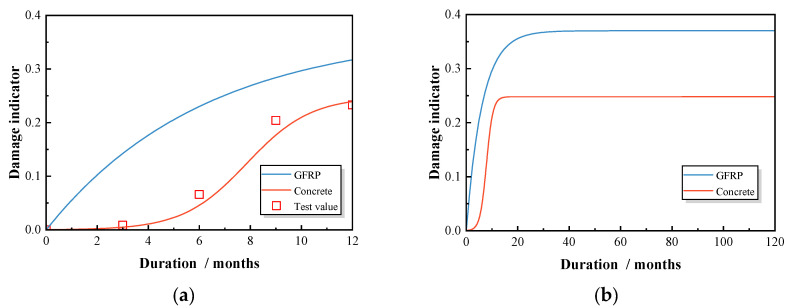
Damage indicator evolution for GFRP and concrete: (**a**) short-term damage evolution; (**b**) long-term damage evolution.

The compressive strength of the concrete was measured concurrently with the pull-out test. Since the first exposure period was 3 months, the concrete had already undergone the strengthening phase. Thus, the measurements showed that the compressive strength of concrete continuously decreased with the increase in exposure time, as indicated in Figure 13. The relationship between concrete damage and erosion time can be described using the Boltzmann equation [32], as shown in Equation (20). The Boltzmann equation is characterized by stability in the early stage, rapid changes in the medium term, and stability again in the later stage. Additionally, it is capable of representing any changes induced by parameters, as the system will return to a new equilibrium state following any disturbance applied to it [33,34]. Here, the damage indicator $d_{c}$ can be derived from the fitted test data with the following Equation (20):

(20) $$d_{c}=a_{1}-\frac{a_{1}-a_{2}}{1+exp\left(\frac{t-a_{t}}{d_{x}}\right)}$$

where t is the duration of chloride exposure in months; $a_{1}$, $a_{2}$, $d_{x}$, and $a_{t}$ are fitting parameters between damage and erosion time. In this experiment, these parameters were fitted as $a_{1}=0.25$, $a_{2}=0$, $d_{x}=1.26$, and $a_{t}=7.86$, utilizing the measurements of concrete compressive strength displayed in Figure 17a.

For the GFRP bars, for ease of calculation, a constant degradation mechanism is assumed, unaffected by variations in time and temperature [35]. The damage factor of the elastic modulus for the GFRP bar ($d_{r}$) can be determined using the established Arrhenius model [36], expressed as:

(21) $$d_{r}=\frac{\alpha }{\beta }\cdot \frac{1-exp(-\beta t)}{100}$$

(22) $$\beta =\frac{\left(14.0-pH\right)/pH}{\frac{T_{c}}{T_{r}}\cdot \phi_{F}\cdot {\left(\frac{2r}{D_{r}}\right)}^{2}}$$

where α is a calculation constant, set to 0.2 in this paper; β is reduction factor for GFRP degradation; pH is the pH value within the concrete, fixed at 13.6 for normal concrete; $T_{c}$ denotes the current temperature of the GFRP bars in Kelvin; $T_{r}$ is the reference temperature, set to 293 K; $D_{r}$ is the reference value, established at 2.0 mm; $\phi_{F}$ is the surface coefficient quantifying the contact area of GFRP bars with the surrounding solution (for a GFRP bar placed in pure solution, this value is 1.0, while for GFRP bars in concrete, this value is defined as the porosity of cement paste surrounding GFRP bars).

The evolution of the damage indicators $d_{r}$ and $d_{c}$ is illustrated in Figure 17, highlighting the deterioration characteristics under chloride wet–dry cycles. The $d_{r}$ curve exhibits a steep slope during the first three months, indicating rapid damage accumulation in the GFRP bar. After this period, the slope decreases, suggesting a transition to a slower damage phase, likely due to the saturation of chloride ion penetration, which reduces the overall diffusion rate and stabilizes surface damage [36]. In the subsequent months, the damage rate remains relatively stable with minimal fluctuations. In contrast, the $d_{c}$ curve for concrete damage follows an S-shaped pattern. Initially, the damage rate is low, with a gentle slope during the first three months. Between months three and eight, the damage accumulation accelerates significantly, as reflected by the increased slope. After the ninth month, the damage rate slows down, and the curve flattens, indicating that the concrete has entered a more stable phase of damage accumulation.

These two damage indicators, $d_{r}$ and $d_{c}$, can be utilized to refine the analytical model presented in Section 4.1. The long-term elastic modulus of GFRP, $E_{r}^{\prime }$, and the concrete compressive strength, $f_{c}^{\prime }$, under chloride exposure can be expressed as follows:

(23) $$E_{r}^{\prime }=(1-d_{r})E_{r}$$

(24) $$f_{c}^{\prime }=(1-d_{c})f_{c}$$

This modification allows for the prediction of bond–slip curves across various chloride exposure durations. However, it is important to note that the relatively short exposure duration in the experiments may limit the predictive model’s accuracy regarding long-term performance. Additional experimental data are necessary to further refine and enhance the calculation model.

### 4.3. Model Validation

**Figure 18:**
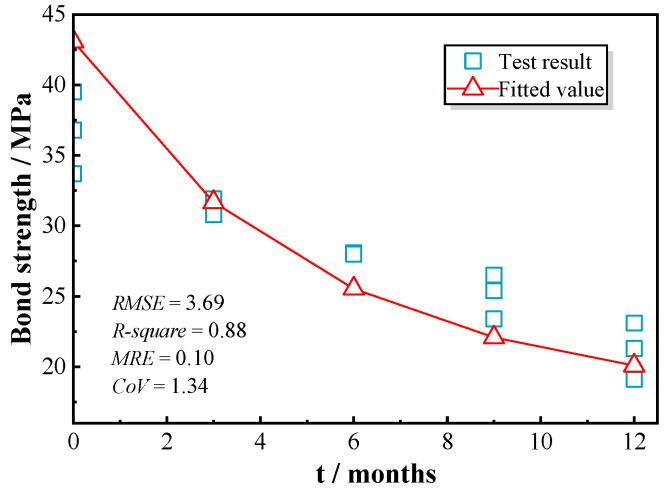
Relationship between coefficient k and test results.

Based on the analysis derived in Section 4.1 and Section 4.2, a predictive method was developed to estimate the bond–slip behavior between GFRP bars and concrete under the influence of chloride wet–dry cycles. The variable, $u_{s}$, representing the friction coefficient at the interface between GFRP bars and concrete was set to 0.5 for the RG series and 0.55 for the SG series [37,38]. The ratio of the transverse elastic modulus of GFRP to its longitudinal elastic modulus (γ) was selected as 0.25 for RG series and 0.125 for SG series in this paper [30]. The coefficient k, obtained through the regression of experimental results, was determined to be 0.85, as shown in Figure 18.

According to ACI 318 (2012) [39], the shear modulus of concrete can be calculated as:

(25) $$E_{c}=\frac{57,000\sqrt{\left({145\times f}_{c}\right)}}{290\times \left(1+0.2\right)}$$

where $f_{c}$ is cylinder compressive strength, which can be assumed to be 0.8 times the prism compressive strength in this test.

**Figure 19:**
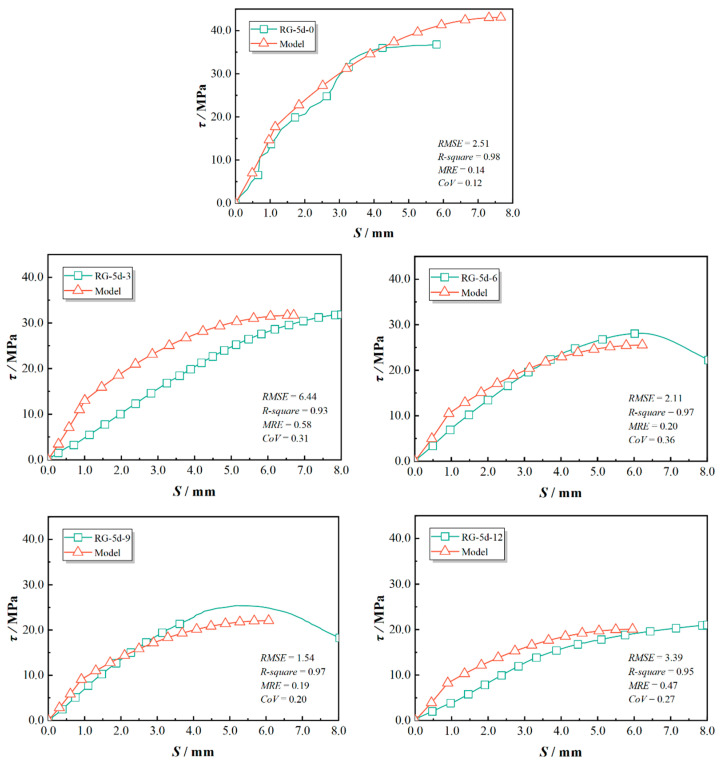
Prediction accuracy for RG-5d series specimens.

**Figure 20:**
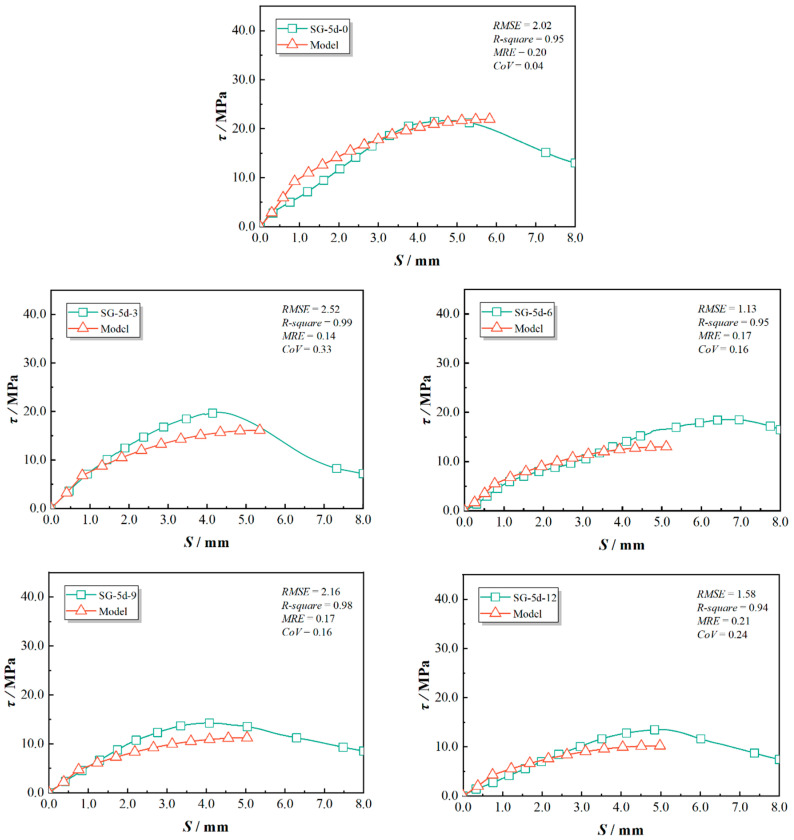
Prediction accuracy for SG-5d series specimens.

**Figure 21:**
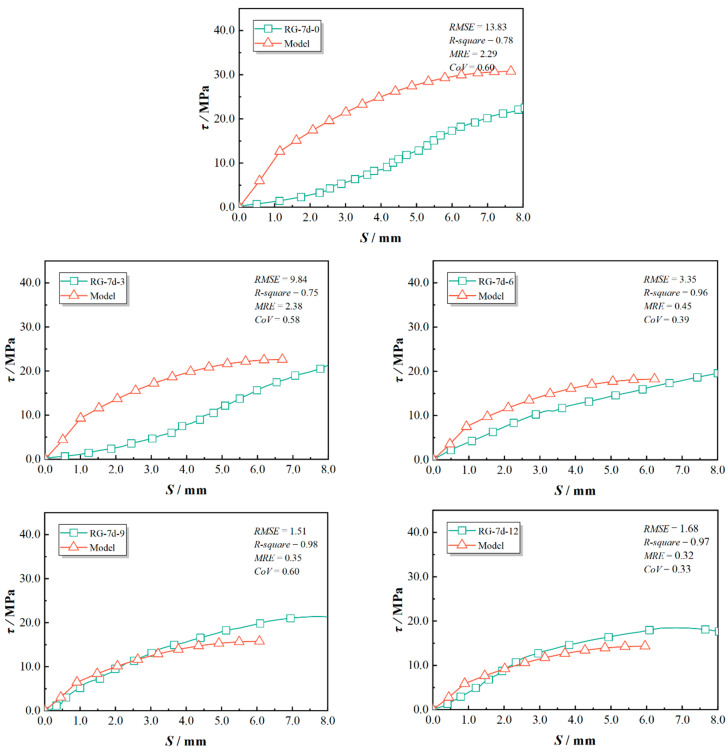
Prediction accuracy for RG-7d series specimens.

The predicted bond–slip curves and the statistical evaluations were compared with the experimental results and are presented in Figure 19, Figure 20 and Figure 21. This study used *RMSE*, *R-square*, *MRE*, and *CoV* to quantify the feasibility of the model. Specifically, a smaller *RMSE* indicates better model performance; an *R-square* above 0.8 typically suggests that the model fits the data well; an *MRE* less than 0.2 is considered a good level; and a *CoV* below 0.3 indicates superior predictive performance. As indicated in Figure 19, Figure 20 and Figure 21, the proposed model showed good agreement for test specimens exhibiting failure pattern B. In contrast, specimens with failure pattern A (specimens RG-7d-0, RG-7d-3, and RG-7d-6) demonstrated greater variability in the correlation metrics. This discrepancy is primarily due to the assumption in the proposed analytical model, which does not account for concrete crushing damage.

Table 5 presents a comparison of the predicted and tested peak values for the GFRP bar pull-out tests in concrete. The ratio of predicted bond strength ($\tau_{p}$) to the tested strength ($\tau_{t}$) was close to 1 and exceeded 0.7 for all tested specimens, indicating a strong predictive capacity of the proposed analytical model for assessing the ascending portion of the bond–slip performance. For the RG-5d group, the ratio of predicted to experimental peak strength ($\tau_{p}/\tau_{t}$) ranged from 0.87 to 1.17, with an average error of 2%. The ratio of predicted to experimental peak slip ($s_{p}/s_{t}$) varied between 0.69 and 1.32, with an average error of 1%. Notably, the highest prediction errors for both peak strength and peak slip in the RG-5d group occurred in the RG-5d-0 test, and they were attributed to a different failure mode. It was possible for local concrete crushing in specimen RG-5d-0 to result in a lower measured peak strength than that predicted for GFRP rib failure.

In the SG-5d group, the $\tau_{p}/\tau_{t}$ ratio ranged from 0.70 to 1.01, with an average error of 19%. The $s_{p}/s_{t}$ ratio spanned from 0.77 to 1.26, with an average error of 11%. It is evident that the predicted peak strengths for the SG-5d group were generally lower than the experimental values, which may be due to the conservative selection of the γ value. Additionally, the proposed GFRP damage indicator $d_{r}$ in Equations (22) and (23) did not account for the effects of the sand-blasted surface. Previous studies have indicated that sand-blasted surfaces are more susceptible to damage and stripping from the GFRP bar.

For the RG-7d group, the $\tau_{p}/\tau_{t}$ ratio lay between 0.74 and 1.15, with an average error of 7%, while the $s_{p}/s_{t}$ ratio ranged from 0.56 to 0.95, with an average error of 27%. The predicted peak bond strength for the RG-7d group was calculated using a reduction factor based on the RG-5d group. However, the longer bond length led to a deeper chloride erosion pathway when exposed in the chloride solution tank, potentially enhancing chloride resistance for the RG-7d series pull-out specimens [11]. Furthermore, the $\tau_{p}$ for the RG-7d group was derived under the assumption of a uniform bond stress distribution along the GFRP, similar to that of the RG-5d group. This assumption overlooked the non-uniform distribution of the bond stress, resulting in an overestimation of bond strength [8].

**Figure 22:**
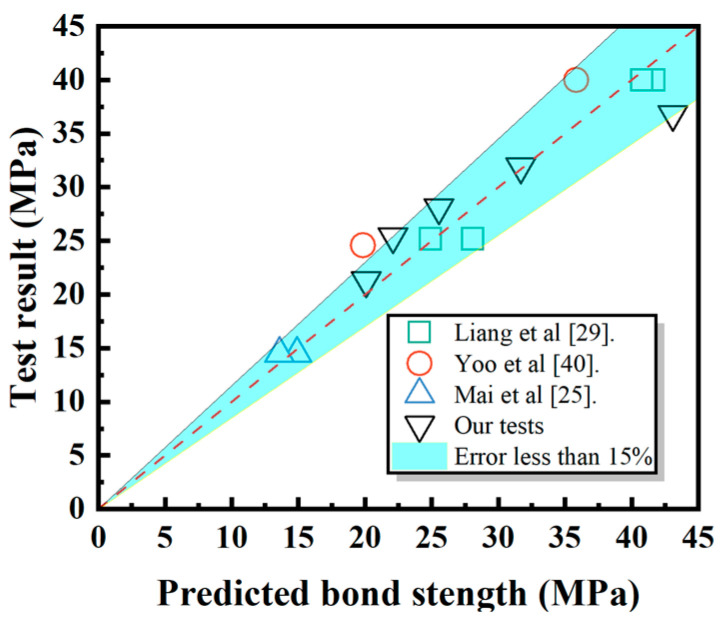
A comparison of bond strengths and predicted results from various sources.

Furthermore, Figure 22 presents a comparison between the bond strength test results from various studies and the predicted values from the model developed in this study [25,29,40]. It can be observed that the predicted bond strengths align well with the experimental results across multiple datasets, demonstrating the reliability of the proposed model. The model provides a highly accurate estimation of bond strength, effectively capturing the influence of key parameters. While some variations exist due to differences in experimental conditions and material properties, the overall trend indicates that the proposed model can robustly predict bond performance in chloride-exposed environments. Therefore, the validation results confirm that the model developed in this study exhibits sufficient accuracy and applicability for predicting the bond behavior of FRP bars in concrete.

## 5. Model’s Application

### 5.1. Analysis of Parametric Sensitivity

**Figure 23:**
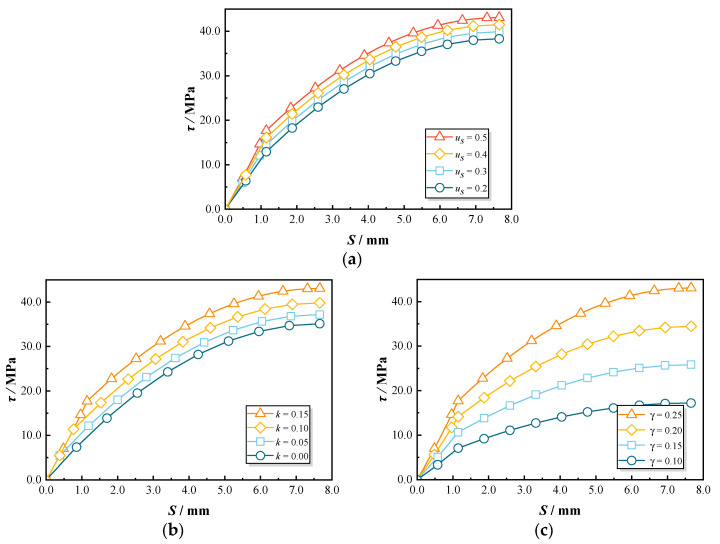
Parametric sensitivity in the predicted bond–slip curve: (**a**) effect of $u_{s}$; (**b**) effect of k; (**c**) effect of γ.

The parameters $u_{s}$, k, and γ are essential for the proposed bond–slip model. This section analyzes the sensitivity of the predicted bond–slip curves to variations in $u_{s}$, k, and γ. Initially, Figure 23a illustrates the effect of different $u_{s}$ values (0.4, 0.3, 0.2, and 0.1) on the bond–slip curves. The parameter $u_{s}$ represents the contribution of friction to the bond performance between GFRP bars and concrete during pull-out testing, where a higher $u_{s}$ value indicates a rougher contact surface and increased frictional resistance. The results show that decreasing $u_{s}$ slightly reduces the convexity of the predicted curve. Although the peak strength is only moderately reduced by approximately 11%, the strength at the end of the elastic stage exhibits a more significant decrease of around 27%. This is because friction plays a dominant role in the initial stages of the pull-out test. As the GFRP bar slip increases, the bond stress at the interface is primarily governed by mechanical interlock rather than friction [7]. Consequently, the friction coefficient $u_{s}$ primarily affects the elastic stage characteristics of the bond–slip curve, demonstrating minimal sensitivity in the subsequent phases.

In this study, the coefficient k influences the elastic stage of the pull-out test, as detailed in Equation (6). To assess the impact of k on the predicted curves, different values of k (1.0, 0.95, 0.90, 0.85) were incorporated into the proposed model for comparison, as illustrated in Figure 23b. As the coefficient k increases, the slip for the elastic stage shortens. It clearly demonstrates that the predicted curve exhibits a diminishing “convex” trend with increasing k. When k changes from 0.85 to 1.00, the peak strength gradually decreases by 19%. These results indicate that the predicted curve shows a certain degree of sensitivity to variations in k.

To investigate the impact of γ on the predicted curve, various γ values were introduced into the proposed model for comparison. Figure 23c illustrates the comparison among γ values of 0.25, 0.20, 0.15, and 0.10. The parameter γ represents the discount factor for the transverse elastic modulus of the GFRP. As γ decreases, the overall trend of the curve flattens, and the peak strength correspondingly reduces. The predictive model employed in this study as based on the elastic deformation of GFRP ribs. For specimens with other identical parameters, a smaller γ value indicates that the same deformation will generate a reduced elastic force. Consequently, the predicted curve declines with the decrease in γ.

### 5.2. Long-Term Bond Performance Prediction

The validated bond–slip prediction model was applied to evaluate the long-term bond performance between GFRP bars and concrete under prolonged chloride exposure. This section examines the progressive changes in the bond–slip behavior of GFRP–concrete pull-out specimens over a 10-year period. It is assumed that concrete degradation follows the trends outlined in Section 4, while GFRP bars are exposed to stable chloride conditions, with similar environmental factors in terms of concentration, temperature, and so on. All parameters used in this analysis are consistent with those defined in Section 4.3.

**Figure 24:**
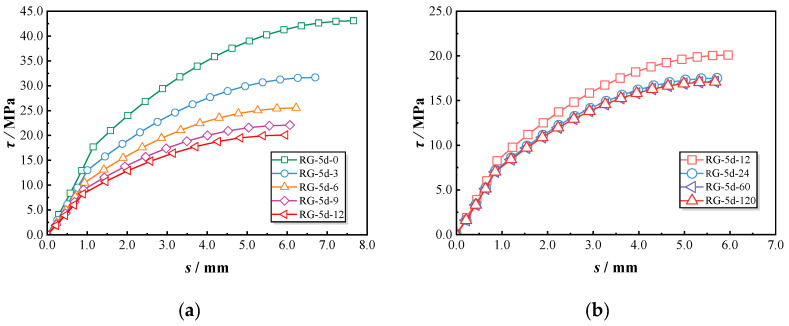
The predicted bond–slip curves for RG-5d specimens. (**a**) Short-term prediction; (**b**) Long-term prediction.

Figure 24a illustrates the evolution of the bond–slip curve for GFRP–concrete (RG-5d) specimens over one year. Significant changes in the bond–slip curves are observed at different intervals, although the rate of strength degradation decreases over time. The peak bond strength declines by approximately 26.5% within the first three months, while the reduction from months 9 to 12 is only around 9%. This indicates that damage accumulation in GFRP–concrete bond specimens is more severe in the early stages under chloride dry–wet cycles.

Figure 24b extends this analysis to a 10-year exposure period. The most pronounced changes in the bond–slip curve occur within the first 24 months, after which minimal alterations are observed beyond 36 months, with peak bond strength stabilizing at 17.1 MPa. These results suggest that the bond performance degradation rate of GFRP–concrete (RG-5d) pull-out specimens diminishes with increased exposure time.

In summary, this study offers theoretical insights into predicting the long-term bond–slip behavior between GFRP bars and concrete under chloride wet–dry cycles. However, the predictive outcomes are based on a mechanical model that may not fully capture complex real-world conditions. Specifically, the model assumes uniform damage distribution within the material, while in practice, damage could be localized and heterogeneous, potentially affecting the accuracy of the bond–slip behavior predictions. Therefore, future research should incorporate additional experimental data to refine and validate the model, thereby enhancing its precision and applicability. It should be noted that this study employs an indoor dry–wet cycle accelerated test, in which the experimental duration is relatively short. While discrepancies between this accelerated testing method and real-world conditions remain, it has been widely adopted by many researchers and has been proven to be a scientifically feasible approach for studying the degradation of the bond performance between GFRP bars and concrete.

## 6. Conclusions

This study systematically investigated the bond behaviors between GFRP bars and concrete under chloride wet–dry cycles, focusing on the effects of bond length and surface texture. The experimental results demonstrate that both bond length and surface texture significantly influence bond performance and degradation patterns during chloride exposure. An analytical bond–slip relation model with high applicability and accuracy was proposed. The main conclusions are summarized as follows:

(1) The application of a sand-coated surface on GFRP bars resulted in significantly lower bond strength and stiffness compared to threaded ribbed GFRP bars. However, the sand-coated GFRP bars exhibited better resistance to deterioration under chloride exposure, with a reduction of approximately 37.7% after 12 months, compared to a 42.1% reduction in threaded ribbed GFRP bars.

(2) Bond length significantly influenced the failure pattern and bond–slip behaviors between GFRP and concrete. Pull-out specimens with shorter bond lengths were more prone to peeling-off failure after exposure, and the failure mode in RG-7d group changed from pull-out failure to peeling-off failure as the exposure duration exceeded 9 months. GFRP bars with a shorter bond length (5*d*) consistently exhibited higher bond strength across all exposure durations, while increasing the bond length to 7*d* resulted in a more gradual decrease in bond strength.

(3) The test results revealed a clear relationship between chloride exposure duration and the degradation of GFRP–concrete bond performance. A comparison of the retentions in concrete compressive strength and bond strength indicated that the deterioration of GFRP bars is a critical factor contributing to bond deterioration.

(4) An analytical model was developed that incorporated GFRP bar geometry, surface texture, and bond length, and it was validated against experimental data. The model successfully predicted bond behavior under varying conditions, demonstrating its effectiveness in capturing the key factors influencing bond performance. This model can also predict bond behavior over a ten-year period under continuous chloride exposure.

(5) Future research will focus on optimizing the predictive model to improve its applicability and accuracy in engineering practice. Additionally, extended experimental studies will be conducted to obtain more comprehensive long-term degradation data, as the current study was limited to 12 months of exposure. These findings will help validate and refine the model, ensuring its reliability in assessing the durability of GFRP-reinforced concrete structures.

## Figures and Tables

**Table 1:** Existing experimental research on GFRP bond performance.

Reference	Chloride Environment		FRP Bar		Prediction Model
	Environment	Duration (Day)	Bond Length	Surface Texture	
Zhang et al. (2024) [5]	Dry–wet cycles	360	5*d*	RB	
Lu et al. (2023) [6]	Emersion	180	5*d*	RB	Mathematical
Nelson et al. (2024) [7]	/	/	/	/	Mathematical
Shi et al. (2024) [8]	/	/	5*d*/10*d*/15*d*	SM	Mathematical
Zhou et al. (2024) [9]	/	/	5*d*	SC/RB	Mathematical
Chen et al. (2023) [10]	/	/	5*d*	RB	/
Hussain et al. (2022) [11]	Emersion	90	5*d*/10*d*/15*d*	RB	/
Yang et al. (2022) [12]	/	/	3*d*/5*d*	RB	Mechanical

Note: *d* denotes the diameter of the tested FRP bars. SM, SC, and RB refer to the surface textures of the FRP bars: smooth, sand-coated, and ribbed, respectively [5,6,7,8,9,10,11,12].

**Table 2:** Concrete admixture design (unit: kg (for one m^3^ of concrete)).

Strength Grade	Water–Cement Ratio	Water	Cement	Sand	Stones
C40	0.49	220	449	615	1116

**Table 3:** Design parameters for the tested pull-out specimens.

Group	Specimen	Surface Texture	Diameter	Bond Length	Chloride Duration
RG-5d	RG-5d-0	Threaded ribbed	12 mm	5*d*	0 months
	RG-5d-3	Threaded ribbed	12 mm	5*d*	3 months
	RG-5d-6	Threaded ribbed	12 mm	5*d*	6 months
	RG-5d-9	Threaded ribbed	12 mm	5*d*	9 months
	RG-5d-12	Threaded ribbed	12 mm	5*d*	12 months
SG-5d	SG-5d-0	Sand-coated	12 mm	5*d*	0 months
	SG-5d-3	Sand-coated	12 mm	5*d*	3 months
	SG-5d-6	Sand-coated	12 mm	5*d*	6 months
	SG-5d-9	Sand-coated	12 mm	5*d*	9 months
	SG-5d-12	Sand-coated	12 mm	5*d*	12 months
RG-7d	RG-7d-0	Threaded ribbed	12 mm	7*d*	0 months
	RG-7d-3	Threaded ribbed	12 mm	7*d*	3 months
	RG-7d-6	Threaded ribbed	12 mm	7*d*	6 months
	RG-7d-9	Threaded ribbed	12 mm	7*d*	9 months
	RG-7d-12	Threaded ribbed	12 mm	7*d*	12 months

**Table 4:** Failure modes and peak characteristics of tested pull-out specimens.

Specimen	Failure Patterns	$\tau_{t}$		$s_{t}$	
		Value/MPa	Rate/%	Value/mm	Rate/%
RG-5d-0	A	36.8	100.0	5.8	100.0
RG-5d-3	B	31.9	86.7	8.1	139.7
RG-5d-6	B	28.1	76.4	6.2	106.9
RG-5d-9	B	25.4	69.0	5.1	87.9
RG-5d-12	B	21.3	57.9	8.6	148.3
SG-5d-0	B	21.7	100.0	4.7	100.0
SG-5d-3	B	19.8	91.2	4.2	89.4
SG-5d-6	B	18.6	85.7	6.6	140.4
SG-5d-9	B	14.3	65.9	4.0	85.1
SG-5d-12	B	13.5	62.2	4.9	104.3
RG-7d-0	A	29.0	100.0	10.7	100.0
RG-7d-3	A	25.6	88.3	11.9	111.2
RG-7d-6	A	22.2	76.6	10.4	97.2
RG-7d-9	B	21.4	73.8	7.6	71.0
RG-7d-12	B	18.5	63.8	6.8	63.6

**Table 5:** Comparison of the predicted and tested peak points.

Specimen	Failure Patterns	$\tau_{P}$	${\tau_{P}/\tau }_{t}$		$s_{P}$	${s_{P}/s}_{t}$	
			Rate	Average		Rate	Average
RG-5d-0	A	43.08	1.17	0.98	7.66	1.32	1.01
RG-5d-3	B	31.68	0.99		6.70	0.83	
RG-5d-6	B	25.54	0.91		6.22	1.00	
RG-5d-9	B	22.09	0.87		6.07	1.19	
RG-5d-12	B	20.09	0.94		5.96	0.69	
SG-5d-0	B	21.94	1.01	0.81	5.83	1.24	1.11
SG-5d-3	B	16.13	0.81		5.35	1.27	
SG-5d-6	B	13.01	0.70		5.11	0.77	
SG-5d-9	B	11.25	0.79		5.04	1.26	
SG-5d-12	B	10.23	0.76		4.98	1.02	
RG-7d-0	A	30.77	1.06	0.93	7.66	0.72	0.73
RG-7d-3	A	22.63	0.88		6.70	0.56	
RG-7d-6	A	18.24	0.82		6.22	0.60	
RG-7d-9	B	15.78	0.74		6.07	0.80	
RG-7d-12	B	21.2	1.15		6.43	0.95	

## Data Availability

Data are contained within the article.
